# Multilayer homogeneous dielectric filler for electromagnetic invisibility

**DOI:** 10.1038/s41598-018-32070-5

**Published:** 2018-09-17

**Authors:** Alberto Serna, Luis J. Molina, Javier Rivero, Luis Landesa, José M. Taboada

**Affiliations:** 1 University of Extremadura, Departamento de Tecnología de los Computadores y las Comunicaciones, Cáceres, 10003 Spain; 2 Istituto Superiore Mario Boella, Torino, Italy

## Abstract

In recent years, invisibility has become a research area of increasing interest due to the advances in material engineering. It may be possible to achieve invisibility through cloaking devices by coating the body using one or more layers of materials with the proper electromagnetic properties. By using techniques associated to plasmonic cloaking it is maybe possible to obtain also invisibility for small objects with several layers of homogeneous materials working from inside the object. We demonstrate numerically that it is, therefore, possible to achieve invisibility through an inner system based on scattering cancellation techniques.

## Introduction

Although there is a wide variety of areas where the term “invisibility” may appear, like acoustics^1–3^, thermodynamics^4^, or even quantum mechanics^5,6^, this work is focused on electromagnetic invisibility in the optical range^7–9^. Ever since Victor Veselago predicted the existence of materials with negative permittivity and permeability in 1967^10^, the development of material science has undergone a fast growth^11^. As a result of this, the study of invisibility passed from being just a problem of the sci-fi novels to become a problem at the modern science.

The “visibility” of an object can be measured, among others concepts, by the scattering cross section (SCS). SCS is defined as a ratio between the total scattered power and the total incident power. Some techniques of invisibilization are based on reducing the SCS close to zero. This means that an invisible object must not reflect any power back to the source neither it must scatter light in other directions and, of course, it must not create a shadow caused by the absorption of power.

Techniques based on transmission lines, transformation optics and plasmonic cloaking have demonstrated important results for narrow-band ideal invisibility.

The transmission lines systems^12–15^ work by conducting the fields through the object, using for it an intricate network within the cloaked region. In order to work properly a transition layer must be applied to the object to couple the incident fields to the transmission-line network. This method may achieve high levels of invisibility.

Transformation optics techniques^16–20^ focus on light ray guide using for it a very specific combination of materials, which perform a coordinate change around the cloaked object based on the anisotropic and non-homogeneous properties of these. This change of coordinates bends the path of light rays around the object, avoiding any interaction between the light and the cloaked area which contains the object. In other words, the object cannot be seen.

On the other hand, plasmonic cloaking based on scattering cancellation^21–29^ pursue a reduction of the scattered fields without relying on ray guide or any kind of coordinate transformation. This allows the use of homogeneous and isotropic materials. Due to the possibility of using this kind of materials, the application of optimization algorithms is feasible for this technique^25,28,29^.

However, up until now all these techniques have relied on applying external additional devices to the target object, which changes the system geometry by making it bigger. Some kind of materials such as liquids, gasses or plasmas are difficult to include in the outer layer of the body. Furthermore, when a system needs to interact with the environment, the external cloak can make it difficult, e.g. a probe or a sensor or a communication system that needs to receive or transmit an electromagnetic wave.

In this paper we explain the invisibility approach through scattering cancellation by filling the object with a scheme of layers of homogeneous and isotropic materials. The use of the filler can overcome some difficulties of outer layers in some systems (e.g. their use in probes or sensors as explained above), but it only works for penetrable objects not much larger than a wavelength and composed by lossless materials.

The proposed methodology for obtaining invisibility through scattering cancellation is based on using Genetic Algorithms for minimizing the electromagnetic scattering of the original object with the filler inclusion. A similar procedure has been used in^28^ and in^29^ where anisotropic external multilayer cloak has been optimized and used in experimental tests. In the present work, valid for 3D structures, we use the same concept of multilayer cloak as in^28,29^, but only for isotropic materials. The main difference is the use of an inner cloak instead an external cloak. In general, an inner cloak does not obtain the same level of invisibility than multilayer external cloaks obtained in previous works. The most important interface in the scattering of the object was the most external, and this make it difficult to obtain the same level of invisibilization that an external cloak. It is clear that a filler can not invisibilize nonpenetrable objects because the filler did not receive electromagnetic fields. In fact, PEC objects as used in^28,29^ are impossible to invisibilize with inner cloaks.

The concept of filling the objects with homogeneous dielectrics was used in the mirage systems^30^, which are not intended for invisibility, but to “change” the way the object looks to an observer. The concept of filling was used also in the “anti-cloak”^31,32^ where a filler is used to undo the cloaking effect; in this case the filler is included between the cloak and the object.

In the following sections the scattering cancellation filler is explained, results are introduced as well as the method to calculate the constitutive parameters of each layer of material.

## Invisibility Multilayer Filler

Whereas in transformation optics techniques any interaction between the cloaked device and the impinging light is avoided, the scattering cancellation techniques, on which this approach is based, do not impose this condition. This makes possible the application of the concept of an invisibilization device working from within the object. The details are explained in the methods section.

**Figure 1:**
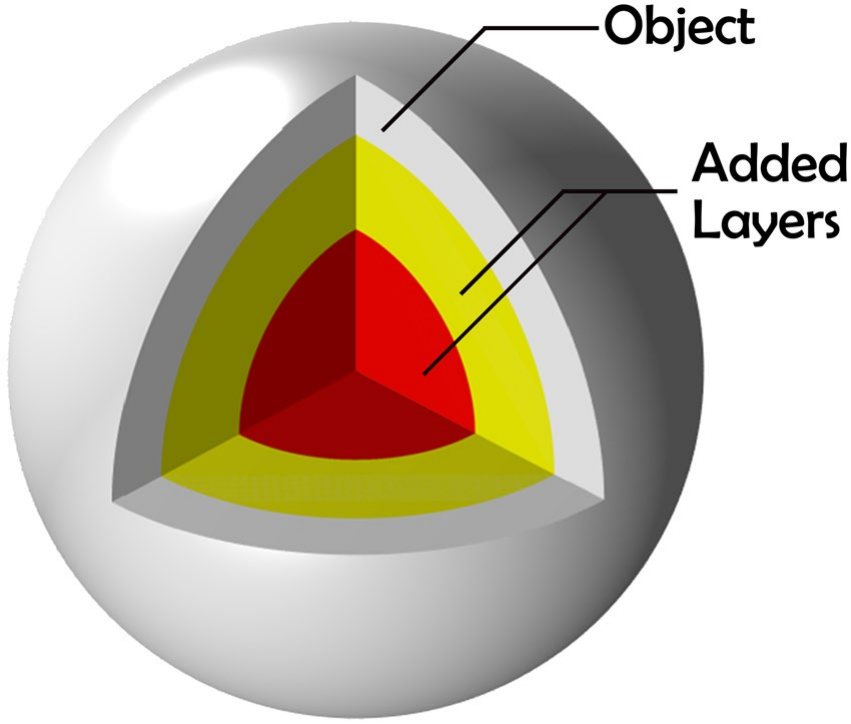
Structure of the multilayer dielectric filler.

To build up the device, as in some cloaking works using scattering cancellation, a multilayer scheme has been used to model the problem. A generic *n*-layer structure scheme used in the optimization process is shown in Fig. 1. The fixed parameters of the problem are the constitutive electromagnetic properties of the body and its size, whereas the parameters to optimize are *ε*_*r*_ and *μ*_*r*_ of each additional layer, as well as their thicknesses, which are limited by the previous layer.

## Results

In order to prove the effectiveness of the inner multilayer homogeneous filler we show three examples of invisibilization of canonical structures of medium size. The three results were obtained by running the optimization process based on Genetic Algorithms for 400 generations, with a population of 128 individuals. All the simulations shown in this work were performed in a cluster with 4 x Xeon E7-8867@2.5 GHz (4 × 16 cores = 64 cores) and 1TB of RAM memory. In all the examples, the objects to be invisibilized are canonical geometries composed of silica (*ε*_*r*_ = 2.1756 + *j*2.36·10^−7^ in the optical regime)^33^ illuminated by a $\hat{x}$ linear-polarized planewave impinging from *θ* = 0. Two of the examples explained correspond to hollow spheres because it is easy to verify the soundness of the solution using Mie series^34^.

The first example corresponds to a sphere of *λ*_0_/2 of diameter and a thickness of 0.05*λ*_0_ filled with a three-layer scheme, being *λ*_0_ the wavelength in free space. The resulting optimized values from the closest layer to the body to the center of the system are *ε*_*r*1_ = 10.39, ${\mu }_{r1}=9.20$, ${\varepsilon }_{r2}=-\,13.50$, ${\mu }_{r2}=-\,7.48$, ${\varepsilon }_{r3}=4.96$ and ${\mu }_{r3}=4.30$, with thicknesses of ${t}_{1}=0.0598{\lambda }_{0}$ and ${t}_{2}=0.0698{\lambda }_{0}$ respectively.

**Figure 2:**
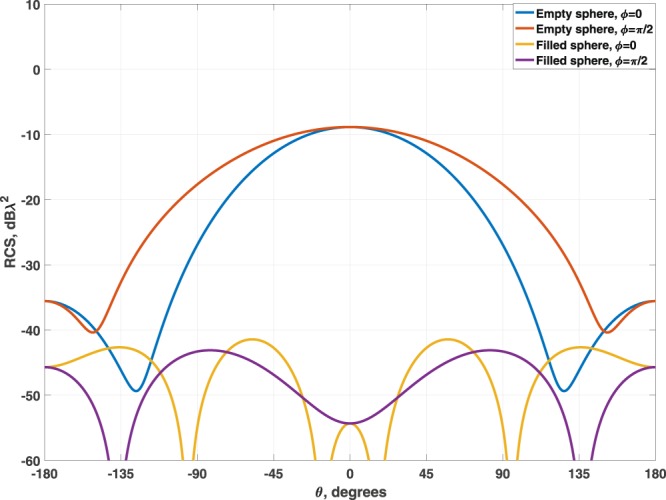
Comparison of the SCS for a silica sphere of *λ*_0_/2-diameter empty and with the multilayer dielectric filler.

Notice that *t*_3_ is equal to the rest of the available space. Figure 2 shows the scattering diagrams for both the empty and the filled structure. A reduction higher that 30 dB in the backscattering is achieved using the multilayer dielectric filler.

**Figure 3:**
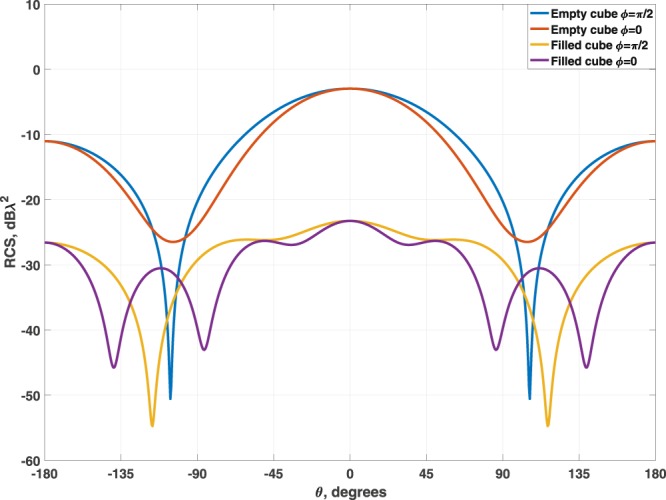
Comparison of the SCS for a silica cube of *λ*_0_/2-side empty and with the multilayer dielectric filler.

The second example corresponds to a cube of *λ*_0_/2 of side and a thickness of 0.05*λ*_0_ in the thinnest point between interfaces filled with a two-layer system. The values obtained after performing the optimization process from the closest layer of the body to the center of the system were ${\varepsilon }_{r1}=-\,3.11$, ${\mu }_{r1}=1.77$, ${\varepsilon }_{r2}=0.98$ and ${\mu }_{r2}=0.54$, with thickness of ${t}_{1}=0.0242{\lambda }_{0}$. The resulting scattering diagram is shown in Fig. 3.

**Figure 4:**
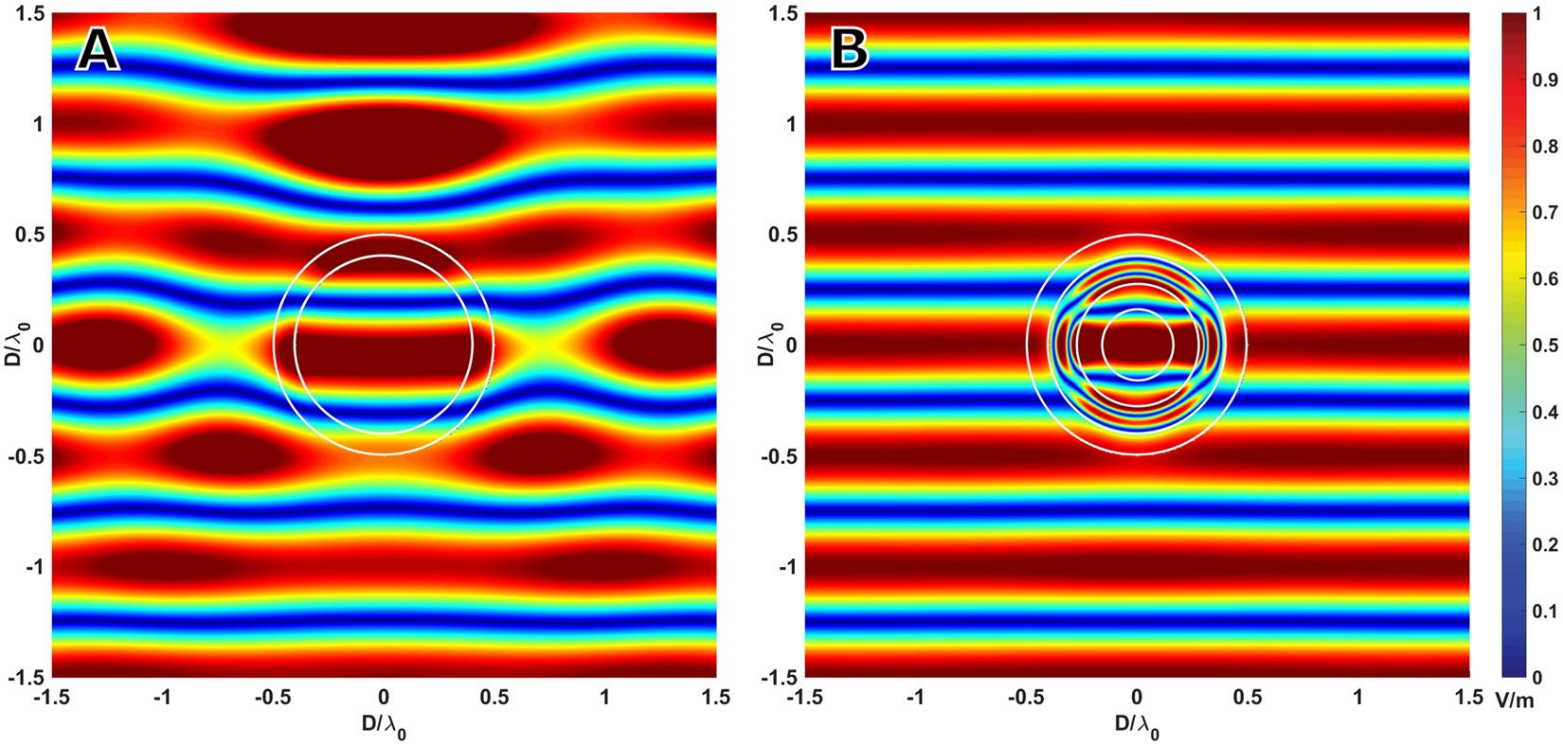
Total electric near field produced by a planewave impinging from the front on (**A**) empty and (**B**) filled spheres.

**Figure 5:**
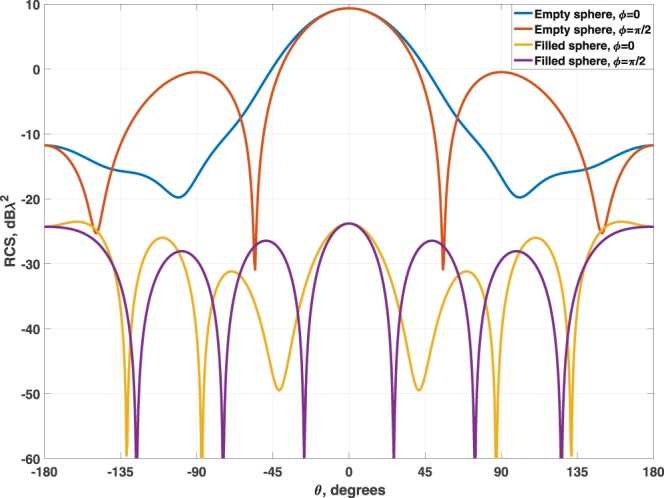
Comparison of the SCS for a silica sphere of *λ*_0_ diameter empty and with the positive multilayer dielectric filler.

Finally, the last example shown in this work corresponds to a sphere of *λ*_0_ of diameter and a thickness of 0.1*λ*_0_ filled with a three-layer dielectric composed of materials with only positive parameters (*ε*, *μ* ≥ 1). The parameters of the multilayer filler are ${\varepsilon }_{r1}=12.61$, ${\mu }_{r1}=4.38$, ${\varepsilon }_{r2}=1.33$, ${\mu }_{r2}=1.44$, ${\varepsilon }_{r3}=2.48$ and ${\mu }_{r3}=1.15$, with thicknesses of ${t}_{1}=0.1233{\lambda }_{0}$ and ${t}_{2}=0.1111{\lambda }_{0}$, which achieves a SCS reduction higher than 27 dB. Figure 4 shows the total electric near field for the invisibilized and the empty sphere produced by the impinging planewave. It can be observed how the fields suffer almost no perturbation despite the presence of the object. In Fig. 5 the scattering diagrams for both the empty and the filled structure can be seen.

## Methods

### Scattering Cross Section

The scattering cross section (SCS) provides a precise way to quantify the power dispersed by the body. A definition of the SCS can be found in (1) when the object is illuminated by a plane wave, where the SCS is denoted as *σ*:

1 $$\sigma (\theta ,\varphi )=4\pi {r}^{2}\frac{|{\overrightarrow{E}}_{s}(\theta ,\varphi {)|}^{2}}{|{\overrightarrow{E}}_{i}(\theta ,\varphi {)|}^{2}}$$

where ${\overrightarrow{E}}_{i}$ is the incident electric field and ${\overrightarrow{E}}_{s}$ is the scattered electric field at a point (*r*, *θ*, *ϕ*).

For measuring the total performance of the SCS, the integral over the solid angle is calculated applying the next equation:

2 $$\overline{{\rm{\Sigma }}}=\frac{1}{4\pi }\oint \sigma (\theta ,\varphi )d{\rm{\Omega }}=\frac{1}{4\pi }{\int }_{0}^{2\pi }{\int }_{0}^{\pi }\sigma (\theta ,\varphi )\sin \,\theta d\theta d\varphi $$

A lower SCS integral implies a higher level of invisibilization and therefore a better performance of the filler.

SCS reduction can be interpreted as a cancellation of the total electric dipole moment by a negative polarized vector induced by a cloak/filler^24^. When the dimensions of the object to be invisibilized are not large compared with the wavelength, the SCS is dominated by the dipole electric moment, which ensures that SCS can be reduced^35^ (the magnetic dipole moment is negligible).

### Genetic Algorithms

In order to obtain an invisibilization filler an optimization algorithm has been applied. In particular a parallelized version of the MIT’s genetic algorithm (GA) library GAlib^36^ was used. Genetic Algorithms are based on the optimization of a population by reproduction, competition, selection, mutation and survival operators, and they have been used before to obtain multilayer cloakings^37^.

Each individual of the population contains a set of parameters which describe the properties of the filler that is analyzed, this is, the permittivities and permeabilities of each layer along with their thicknesses.

In the application of the GA to SCS reduction, the SCS integral, defined as $\overline{{\rm{\Sigma }}}$ and calculated as eq. (2), is used as the fitness function.

### Method of Moments for electromagnetic computation

The methods applied to obtain the SCS of each individual were the surface integral equation (SIE) and the method of moments (MoM)–implemented in the computational electromagnetics library *M*^3^-HEMCUVE^38–42^–applying the *PMCHWT* formulation^43^. The SIE-MoM method is capable of analyzing arbitrary structures in both, shape and size, as long as that the discretization meets a minimum condition. To ensure a high level of accuracy in the results we used a triangular mesh discretization of each multilayer surface with more than 200 triangles for square wavelength.

### GA-MoM integration

**Figure 6:**
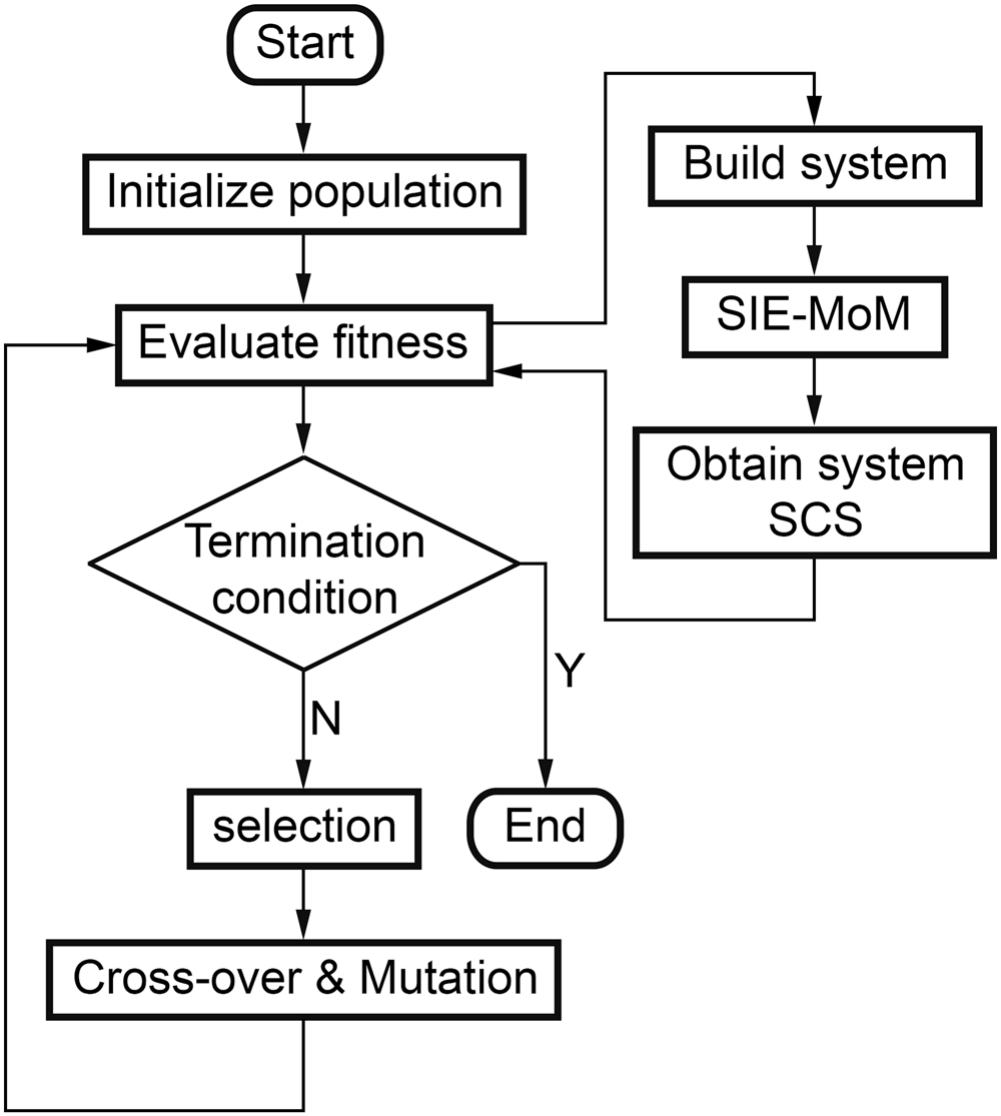
Optimization scheme based on Genetic Algorithms (GA).

The scheme of the whole process of optimization over the SCS is shown in Fig. 6. This scheme was successfully used through *M*^3^-HEMCUVE to perform the optimization of plasmonic antennas^44^.

## Discussion

As it has been presented, the application of the scattering cancellation method makes possible to achieve invisibility from inside an object without using an external device. Also, the concept of working from inside the body opens interesting possibilities for cloaking, because the object can interact with their vicinity without obstruction by the cloak. This cloaking scheme is limited to bodies with empty space inside. Also it is limited to penetrable bodies composed by lossless materials, since the power absorption of the body is unavoidable due to the direct interaction of light with the body. As the invisibilization procedure is based on plasmonic cloaking, it is not possible to obtain invisibility for objects of several wavelengths of size^35^.

Our study was limited to homogeneous and isotropic materials using monochromatic sources. As a matter of fact, with the multilayer dielectric fillers the invisibility presented a very narrow bandwidth. The method has been also tested using only positive dielectric materials with moderate efficiency loss.

The filler is not intended to replace an external cloak, but it is an alternative approach to get invisibility when the inclusion of an external device can disturb the interaction of the target with the environment. In fact, external cloaks analyzed in previous works obtained, in general, a better performance (in addition to using less exotic materials less hard to implement) compared to the results of our paper.
